# Colonization of long term care facility patients with MDR-Gram-negatives during an *Acinetobacter baumannii* outbreak

**DOI:** 10.1186/s13756-017-0209-9

**Published:** 2017-05-16

**Authors:** Ines Zollner-Schwetz, Elisabeth Zechner, Elisabeth Ullrich, Josefa Luxner, Christian Pux, Gerald Pichler, Walter Schippinger, Robert Krause, Eva Leitner

**Affiliations:** 1 Department of Internal Medicine, Section of Infectious Diseases and Tropical Medicine, Medical University of Graz, Auenbruggerplatz 15, A-8036 Graz, Austria; 2 Institute of Hygiene, Microbiology and Environmental Medicine, Medical University of Graz, Graz, Austria; 3 Geriatric Health Centers of the City of Graz, Graz, Austria

**Keywords:** Acinetobacter Baumannii, Colonization, Long term care facility, Disorder of consciousness

## Abstract

**Background:**

We aimed to determine the prevalence of colonization by multidrug-resistant Gram-negative bacteria including ESBL-producing enterobacteriaceae, carbapenem-resistant enterobacteriaceae, *Pseudomonas aeruginosa* and *Acinetobacter baumannii* at two wards caring long term for patients with disorder of consciousness at the Geriatric Health Centers Graz, Austria. During our study we detected two *A. baumannii* outbreaks.

**Methods:**

In August 2015, we conducted a point-prevalence study. Inguinal and perianal swabs were taken from 38 patients and screened for multidrug-resistant Gram-negative rods using standard procedures. Six months after the initial investigation all patients were sampled again and use of antibiotics during the past 6 months and mortality was registered. Genetic relatedness of bacteria was evaluated by DiversiLab system.

**Results:**

Fifty percent of patients were colonized by multidrug-resistant Gram-negative isolates. Five patients harboured ESBL-producing enterobacteriaceae. No carbapenem-resistant enterobacteriaceae were detected. 13/38 patients were colonized by *A. baumannii* isolates (resistant to ciprofloxacin but susceptible to carbapenems). There was a significant difference in the prevalence of colonization by *A. baumannii* between ward 2 and ward 1 (60% vs. 5.6%, *p* < 0.001). Two clusters of *A. baumannii* isolates were identified including one isolate detected on a chair in a patient’s room.

**Conclusions:**

We detected a high prevalence of two multidrug-resistant *A. baumannii* strains in patients with disorder of consciousness at a LTCF in Graz, Austria. Our findings strongly suggest nosocomial cross-transmission between patients. An active surveillance strategy is warranted to avoid missing newly emerging pathogens.

## Background

Long term care facilities (LTCF) play an essential role in contemporary healthcare systems due to an ageing population in the industrialized world. There is increasing evidence suggesting that residents in LTCFs are frequently colonized by multidrug-resistant Gram-negative bacteria [1–3]. Asymptomatic carriage of multidrug-resistant Gram-negative pathogens constitutes a potential source of transmission to other patients. In addition, there is an increased risk of subsequent infection by the multidrug resistant organism [4].

Several organisms are of concern in this setting in particular carbapenemase-producing enterobacteriaceae as well as *Acinetobacter baumannii. A. baumannii* has been shown to colonize the skin [5] as well as abiotic surfaces like equipment used in ICUs [6]. The ability of *A. baumannii* to form biofilms is thought to be pivotal for this colonization [6]. Several studies have demonstrated that *A. baumannii* colonizes patients in LTCFs [3, 7, 8].

The aim of our study was to determine the prevalence of colonization by multidrug-resistant Gram-negative bacteria including ESBL-producing enterobacteriaceae, carbapenem-resistant enterobacteriaceae, *P. aeruginosa* und *A. baumannii* at two wards caring long term for patients with disorders of consciousness (unresponsive wakefulness syndrome and minimally conscious state) at the Geriatric Health Centers Graz, Austria. In the course of this study, an outbreak of *A. baumannii* was discovered and analysed. The results of this analysis are also described in this manuscript.

## Methods

### Setting and study design

We conducted a point-prevalence study in August 2015 at two wards caring long term for patients with disorders of consciousness (unresponsive wakefulness syndrome and minimally conscious state) at the Geriatric Health Centers Graz, Austria. Patients are managed in single or double rooms. The two wards (23 beds each) are located in the same building and are staffed by the same team of health care personnel. Two swabs (Copan, Brescia, Italy) were taken from the perianal region and from skin of the inguinal region (pooled from both sides), respectively. Microbiological sampling was repeated 6 months later in February 2016. Healthcare workers were trained how to obtain microbiological swabs. Sampling was scheduled during the morning ward round before bathing and dressing the patients. Informed written consent was obtained from the legal representatives of all patients.

### Data collection

At the initial survey, structured questionnaires were completed for each patient to document demographic and administrative data as well as data concerning possible risk factors for asymptomatic colonization by multidrug-resistant Gram-negative bacteria. Collected variables included: age, gender, length of stay in the facility, bowel and bladder incontinence, previous hospitalisation or surgery (in past 3 months), previous antibiotic use (in the past 3 months), presence of enteral feeding tubes, tracheostomy and/or urinary catheters, and presence of chronic wounds (decubitus, surgical wounds, and chronic vascular ulcers). Questionnaires for the follow-up survey after 6 months included antibiotic treatment in the past 6 months.

### Microbiological methods

Microbiological samples were transported immediately to the microbiological laboratory of the Institute of Hygiene, Microbiology and Environmental Medicine, Medical University of Graz. Swabs were plated on ChromID ESBL, Chrom ID Carba Smart and MacConkey agar plates (bioMerieux, Marcy l’Etoile, France). The plates were incubated under aerobic conditions at 36 °C and were evaluated for growth after 24 and 48 h. Suspected colonies were further cultivated on blood agar and identified to species level using the automated Vitek MS system (bioMerieux). Antimicrobial susceptibility was tested using Vitek-2 (card AST-N196 and/or N248) with interpretation of the results according to EUCAST breakpoints. All isolates were stored at −70 °C for analysis of genetic relatedness at the end of the study. Automated repetitive PCR with the DiversiLab system (bioMérieux, Nürtingen, Germany) was performed to determine clonal relationships following manufacturer’s instructions. Isolates with a similarity index >95% were considered related and with a similarity index >97.5% as indistinguishable.

Multidrug-resistance was defined according to the recommendations of the Robert Koch Institute (RKI) in Germany issued in 2012 [9]. Briefly, isolates resistant to 3 out of 4 relevant antimicrobial classes (acylureidopenicillin, 3rd/4th generation cephalosporins, carbapenems, fluoroquinolones) were classified as 3MRGN. Enterobacteriaceae resistant to carbapenems were classified as 4MRGN even if the isolate remains susceptible to one other antibiotic class. Isolates resistant to all 4 classes were classified as 4MRGN.

### Statistical analysis

Quantitative variables were expressed as mean ± standard deviation. For statistical analysis Student’s t-test, Chi Square test and Fisher’s exact test were used as appropriate. A *p*-value of less than 0.05 was considered to indicate statistical significance. The statistical software package SPSS 20.0 (Chicago, IL, USA) was used.

## Results

### Patient characteristics

A total of 46 patients was eligible for the study. 38 patients were included in the study (mean age 58.2 ± 13.6 years, 95% CI: 53.7–62.6 years). Eight patients were not included because of a lack of consent of their legal representatives. 21/38 of included patients were male. The mean duration of stay at the ward was 53.6 ± 58 months, 95% CI: 34.2–72.3 months. All patients had enteral feeding tubes. Three patients had suprapubic urinary catheters. None of the patients had chronic wounds/skin defects. Seventeen patients had tracheostomy. None of the patients required mechanical ventilation. None of the patients had been transferred to an acute care hospital within 3 months prior to the study. Five patients had received antibiotic therapy in the 3 months prior to the study.

### Acinetobacter Baumannii outbreak

13/38 patients were found to be colonized by 3MRGN *A. baumannii* isolates. All of these isolates were resistant to ciprofloxacin but susceptible to carbapenems. One patient was co-colonized by an ESBL-producing *E. coli* isolate and a 3MRGN *A. baumannii* isolate. In addition, 5/38 patients were colonized by *A. baumannii* isolates that remained susceptible to carbapenems and ciprofloxacin and were hence not classified as multidrug-resistant. Overall, 18/38 patients were colonized by any *A. baumannii* isolate. Characteristics of patients colonized by 3MRGN *A. baumannii* compared to non-colonized patients are summarized in Table 1. There was a significant difference in the prevalence of colonization by 3MRGN *A. baumannii* between ward 2 and ward 1 (60% vs. 5.6%, *p* < 0.001). Patients colonized by 3MRGN *A. baumannii* had stayed at the ward significantly longer before the study compared to non-colonized patients (91.4 ± 59 months vs. 33.4 ± 47.3 months, *p* = 0.002).

**Table 1:** Clinical characteristics of 3MRGN *A. baumannii* colonized vs. non-colonized patients

	Colonized *n* = 13	Non-Colonized *n* = 25	p=
Age (years, mean ± SD)	54 ± 17.6	60.4 ± 10.7	0.257
Gender (n)			
Male	11	10	0.015
Female	2	15	
Ward (n)			
1	1	17	<0.001
2	12	8	
Length of stay (months, mean ± SD)	91.4 ± 59	33.4 ± 47.3	0.002
Antibiotic therapy past 3 months (n)	4	3	0.203

**Fig. 1:**
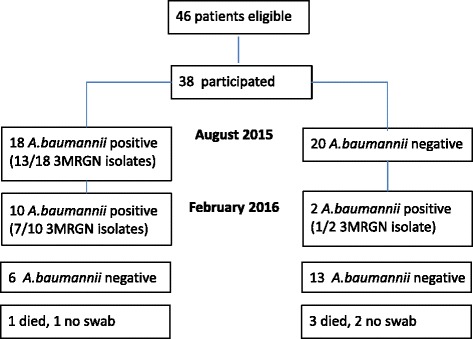
Number of patients colonized by *A. baumannii* isolates in August 2015 and February 2016. 3MRGN: Multidrug-resistance was defined according to the recommendations of the Robert Koch Institute (RKI) in Germany issued in 2012 [9]. Isolates resistant to 3 out of 4 relevant antimicrobial classes (acylureidopenicillin, 3rd/4th generation cephalosporins, carbapenems, and fluoroquinolones) were classified as 3MRGN

Of 18 patients initially colonized by any *A. baumannii* isolate 10 were still colonized after 6 months (in February 2016), whereas 6 patients were not colonized any longer (Fig. 1). There was no significant difference in mortality and antibiotic use between patients colonized by MRGN bacteria compared to non-colonized patients.

As 18/38 of patients were colonized by any *A. baumannii* isolate a source in the environment of ward 2 was suspected. Several studies documenting *A. baumannii* outbreaks demonstrated that water sources such as sinks and the patients’ environment were contaminated by the organism [10–12]. Therefore, swabs were taken from glove boxes, tissue dispensers, sterile filters of a water source, bottles of disinfectant used by cleaning personnel, bedrails, a patient bathtub and a patient elevator into the bathtub, bottles of aromatic oils, sinks in personnel room, a table in personnel room, chairs for visitors in patient rooms. In addition, water drawn from 3 different taps was analysed (source of water for washing patients, kitchen sink, and bathtub). Body care products were not tested as they are used on a single-patient basis. *A. baumannii* was detected on the patient elevator into bathtub (isolate not available for further analysis) and from a chair for visitors in a patient room. The latter was classified as a 3MRGN *A. baumannii* and was included in the Diversilab study. In addition, patients’ charts were reviewed to identify clinical *A. baumannii* isolates during the study period.

### Prevalence of MRGN bacteria

At the initial survey in August 2015, we detected 19/38 patients harbouring MRGN isolates (overall prevalence 50%, Table 2). Five patients were colonized by 3MRGN enterobacteriaceae (3 by ESBL-producing *E. coli* isolates, 2 by ESBL-producing *Klebsiella pneumoniae* isolates). No carbapenem-resistant enterobacteriaceae were detected. Two patients were colonized by 4MRGN *Pseudomonas aeruginosa* isolates.

**Table 2:** Prevalence of colonization by MRGN bacteria

	August 2015	February 2016
	Number of patients	Number of patients
*E. coli* (3MRGN)	3^a^	6^b^
*K. pneumoniae* (3MRGN)	2	0
*P. aeruginosa* (4MRGN)	2	0
*A. baumannii* (3MRGN)	13^a^	7

^a^One patient was co-colonized by *E. coli* (3MRGN) and *A. baumannii* (3MRGN)

^b^Three patients were found to be newly colonized by *E. coli* (3MRGN) in February 2016. Four patients died during the study period. No swabs were received from three additional patients

The follow-up survey was conducted in February 2016. Four patients died during the study period. No swabs were received from 3 patients. Of 5 patients initially colonized by 3MRGN enterobacteriaceae 3 were still colonized, 1 patient was negative, 1 patient had died during the study period. In contrast, 3 patients were newly colonized by 3MRGN enterobacteriaceae at 6 months. Of 2 patients colonized by 4MRGN *P. aeruginosa* at the beginning of the study, one was negative at 6 months and one patient had died. No patient was newly colonized by 4MRGN *P. aeruginosa* (Table 2)*.* There was no significant difference in mortality and antibiotic use between patients colonized by MRGN bacteria compared to non-colonized patients.

### Genetic relatedness

**Fig. 2:**
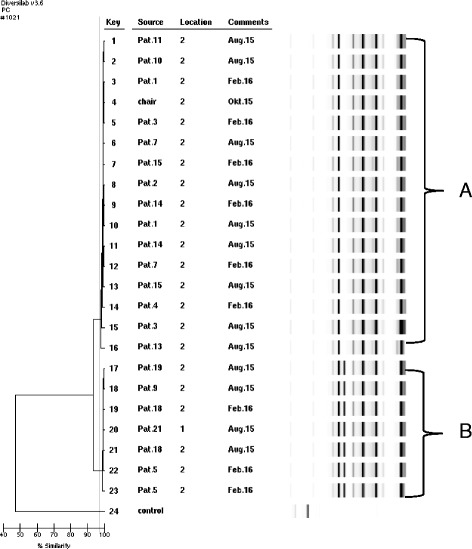
Genetic relatedness of 23 *A. baumannii* isolates by Diversilab System Isolates with a similarity index >95% were considered related and with a similarity index >97.5% as indistinguishable. The *grey line* indicates 97.5% similarity. Location: ward 1 or ward 2. Two clusters of *A. baumannii *isolates were detected (cluster A and cluster B)

Twenty-one 3MRGN *A. baumannii* isolates from patients were included in the DiversiLab analysis as well as the isolate from the environment (chair). One isolate that remained susceptible to ciprofloxacin was also included (patient 9). Two clusters of identical isolates were identified (cluster A and B; Fig. 2). The isolate from the chair was genetically identical to a total of 15 patient isolates and was part of cluster A.

Ten 3MRGN *E.coli* isolates were included in a separate DiversiLab analysis. Two clusters of identical isolates were detected, comprising 6 and 3 isolates, respectively. One isolate was completely different. At the follow-up study (February 2016) 3 patients were found to be newly colonized by 3MRGN *E.coli* isolates. These isolates were all genetically identical and were part of the cluster of six isolates.

## Discussion

This study analyzed the colonization by multidrug-resistant Gram-negative bacteria in patients with disorders of consciousness at a LTCF in Graz, Austria. Notable findings were (1) the prevalence of colonization by multidrug-resistant (3MRGN) *A. baumannii* was unexpectedly high, in particular among patients in ward 2; (2) two clusters of genetically identical *A. baumannii* isolates were identified, including one isolate from the environment; (3) colonization by *A. baumannii* persisted for 6 months in more than half of patients.

*A. baumannii* has emerged as an important pathogen of healthcare-associated infections in critically ill patients in the ICU setting worldwide [10, 13, 14]. In addition, several studies documented that *A. baumannii* also occurs in patients in LTCFs [3, 7, 8]. In Maryland Thom and colleagues investigated the colonization of patients by *A. baumannii* in LTCFs providing care to mechanically ventilated patients [15]. The authors demonstrated that 63% of patients were colonized by *A. baumannii.* This is comparable to our findings although none of the patients in our study required mechanical ventilation. The high prevalence of colonization by *A. baumannii* could be explained by the fact that 90% of patients are transferred to the LCTF directly from external ICUs. In 2015, one fourth of the newly admitted patients were colonized by a multidrug-resistant pathogen at the time of admission. Although all newly admitted patients are screened for multidrug-resistant pathogens, skin sites were until now only screened for the presence of methicillin-resistant *Staphylococcus aureus*. Screening of the skin for multidrug-resistant Gram-negative rods has been introduced as a consequence of our study (see below).

The prevalence of colonization by *A. baumannii* was unexpectedly high in our study with an accumulation of cases in ward 2. We therefore decided to explore the environment and water sources of this ward to identify a source. *A. baumannii* isolates were detected on the patient elevator into a bathtub (isolate not available for further analysis) and from a chair for visitors in a patient room. The latter isolate was genetically identical to a total of 15 patient isolates. In addition, patients colonized by *A. baumannii* had stayed at the ward significantly longer before the study compared to non-colonized patients. Taken together, these findings suggest that cross-transmission between patients by staff may have taken place.

As a consequence of our findings a multimodal intervention program was introduced on both wards by the infection control staff at the end of the study period. It included consequent reinforcement of standard hygiene precautions and barrier precautions as well as repeated education for all occupational groups also including visitors. Colonized patients were washed with antiseptic soap once a month. In addition, disinfection protocols were reviewed specifically addressing the patients’ environment (including the bathtub and the elevator). The time frame for cleaning personnel on both wards was increased by 1 h per day. Admission screening procedures for new patients were adapted to include multidrug-resistant Gram-negative bacteria also from skin swabs. All patients on both wards will be screened for multidrug-resistant bacteria twice a year. These measures were evaluated in a follow-up survey by the infection control staff in December 2016. Only 4/22 patients (18%) in ward 2 were still colonized by 3MRGN *A. baumannii.* The long-term effects of these measures will be evaluated by a follow-up study.

Of note *A. baumannii* was identified from a clinical sample only once during the entire study period, indicating that our patients were in fact only colonized but not infected by *A. baumannii*. Using only clinical samples for surveillance purposes would have underestimated the true prevalence of multidrug-resistant organisms in our setting. Our findings favour the implementation of an active surveillance strategy.

In contrast to the high prevalence of colonization by *A. baumannii* the prevalence of multidrug-resistant enterobacteriaceae was moderate (13%). Our findings are comparable to a French study which reported 10% of nursing home residents to be colonized by ESBL-producing enterobacteriaceae [16]. In contrast to an Italian study, in which colonization by carbapenemase-producing enterobacteriaceae has been described in 6.3% of patients, no carbapenemase-producing enterobacteriaceae were detected [1]. Sixty percent of patients were still colonized by 3MRGN *E.coli* isolates at the time of the follow-up study. Our findings are in line with a study by Birgand et al. investigating the gastrointestinal ESBL-colonization in patients after hospital discharge [17]. Median time to clearance was found to be 6.6 months, ranging from 3.4 to 13.4 months [17]. Three patients were found to be newly colonized by 3MRGN *E.coli* isolates at the time of the follow-up study. These 3 isolates were found to be genetically identical, again pointing to a role of institutional cross-transmission in the spread of multidrug resistant bacteria.

Our study has limitations. To fully assess the prevalence of colonization by *A. baumannii* respiratory samples would probably have been a useful addition. However, our study was designed to assess the prevalence of multidrug-resistant Gram-negative pathogens in general. The high prevalence of *A. baumannii* was unexpected.

## Conclusion

We uncovered two clusters of *A. baumannii* colonizing patients with disorder of consciousness at a LTCF in Graz, Austria. Our findings point toward nosocomial cross-transmission as a cause for these outbreaks. Using only clinical samples for surveillance purposes would have underestimated the true prevalence of *A. baumannii* in our setting. Therefore, an active surveillance strategy is warranted to avoid missing newly emerging pathogens at an early stage. In addition, improved infection control measures are necessary in the LTCF setting.

## Abbreviations

3MRGN: multidrug resistant Gram-negative bacteria according to the definition of Robert Koch Institute
ESBL: extended spectrum betalactamases
EUCAST: European Committee on Antimicrobial Susceptibility Testing
ICU: intensive care unit
LTCF: long term care facility
